# Research on parameters optimization of digital imaging system in red–yellow jadeite color measurement

**DOI:** 10.1038/s41598-022-07715-1

**Published:** 2022-03-07

**Authors:** Ziyuan Liu, Ying Guo, Yanran Shang, Bin Yuan

**Affiliations:** Department of Gemology, China University of Geosciences (Beijing), Beijing, 100083 China

**Keywords:** Other photonics, Solid Earth sciences

## Abstract

The application of digital imaging to color measurement is more and more extensive, which also provides new ideas for gemology. In this paper, the single-factor experiment method and orthogonal experiment design are used to study the influence parameters of the digital imaging system (DIS) on gem color measurement. Among them, the illuminance has the most significant impact on the measurement results, followed by the exposure time, and the change of the measurement distance has an insignificant impact on the results; through range analysis and analysis of variance, the most suitable measurement parameters for red jadeite are 1600 lx, 12,500 μs, 25 cm.

## Introduction

The current methods for evaluating the color of gems include color chips (Munsell, Gem-dialogue), colorimetric stones, Ultraviolet–Visible (UV–Vis) spectrophotometers, and spectrophotometers. Among them, the color chips and the colorimetric stones are used to directly help the eyes to make judgments^1^. These methods are mainly based on the subjective evaluation of the color by the operator, so it takes a lot of time and money to train professional graders. UV–Vis spectrophotometers can provide accurate absorption spectra from which color calculations can be made^2,3^; however, the complex conversion process has hindered their development in gemstone color measurement; hand-held spectrophotometers, as the mainstream gem color measurement tool at present, has been used to measure the color of jadeite green^4^, jadeite red^5,6^, turquoise^7^, peridot^8,9^, amethyst^10^, chrysoprase^11,12^, and extensive gemstone chromatography studies. Because of its convenient operation and reliable data, this tool has already been favored by researchers. But there are also limitations: 1. The size of the gemstone is smaller than the smallest aperture that can be measured by the device, which will lead to significant errors caused by the background. 2. The reading color method is uniform color reading and it’s hard to distinguish and study color-striped gems.

Furthermore, the use of contact measurement in some precious materials like gems may cause unavoidable damage. With the development of color measurement technology in various research fields, such as reproduction and protection of artwork^13^, remote sensing mapping^14^, and dental medicine^15^, non-contact color measurement is widely used. In food science, many experiments and studies have been conducted on quality control^16^. In the fields of geoscience, the non-contact color measurement method is also widely used to classify and sort ores based on colors and textures^17,18^; Oestreich et al. analyzed the composition content of minerals based on digital images^19^; Chatterjee et al. used artificial neural networks to effectively predict the attributes of calcium oxide, alumina, and silica in limestone^20^. Diaz et al. analyzed the defects of contact color measurement in obtaining the color of human tissues and organs, and proposed a non-contact color measurement scheme^21^; Yang et al. analyzed that computer vision after color correction can be more commonly used^22^; In the study of the color of Dunhuang murals, Wan proposed to use the self-made Dunhuang color charts and Color Checker color chips to make ICC feature files to correct the errors generated between different digital cameras, so that the digital protection and dissemination of Dunhuang murals can receive strong technical support^23^; In our other work, experiments by Zhang et al. compared computer vision system and traditional hand-held spectrophotometer for the measurement and evaluation of various colors of jadeite, then judged the applicability by experts panel which makes the color matching subjective^24^; Therefore, in gemology, a digital imaging as one of the non-contact methods can provide researchers with new ideas. In the above-mentioned measurement method, a non-contact measuring device-a spectroradiometer or camera—is used to obtain accurate measurement results under the condition of an external light source.

Gem-quality jadeite is mainly from Burma and Guatemala, which is produced in Jadeitite and has beautiful and durable properties^25,26^. Harlow et al. believe that jadeite is a complex fibrous aggregate of minerals^27^, which gives itself a beautiful, varied appearance, also the reason why it is loved by Chinese people. It is usually colorless and translucent without other impurities. The cause of the color of jadeite can be divided into two types: primary color and secondary color^6,28^; The green of jadeite is the primary color; and the red and yellow is the secondary color, which is caused by exogenous geological activities, and usually produced when impurities, such as water-containing iron oxide, are infiltrated along the cracks and the grain boundaries that are not tightly bound. According to comparative experiments, Wang et al. recognized that the D65 standard light source (representing the average daylight with a color temperature of 6504 K) is the most suitable light source for evaluating Jadeite green^29^; Pan et al., Guo, Li believe that D65 light source is suitable to evaluate the red color of jadeite, tourmaline, and ruby respectively^6,30,31^. The color of jadeite is the most important indicator of value, so it is important to accurately measure and display its color.

However, in our previous study, the effect of objective experimental conditions on the measurement results was not discussed for the scheme of jadeite color measurement. In this paper, we use one single factor method to study how the circumstances of the digital imaging system influence the color measurement and ColorChecker calibration to objectively verify the feasibility of DIS. Based on the Orthogonal Design, the analysis of variance (ANOVA) was used to explore the suitable measurement parameters.

**Figure 1:**
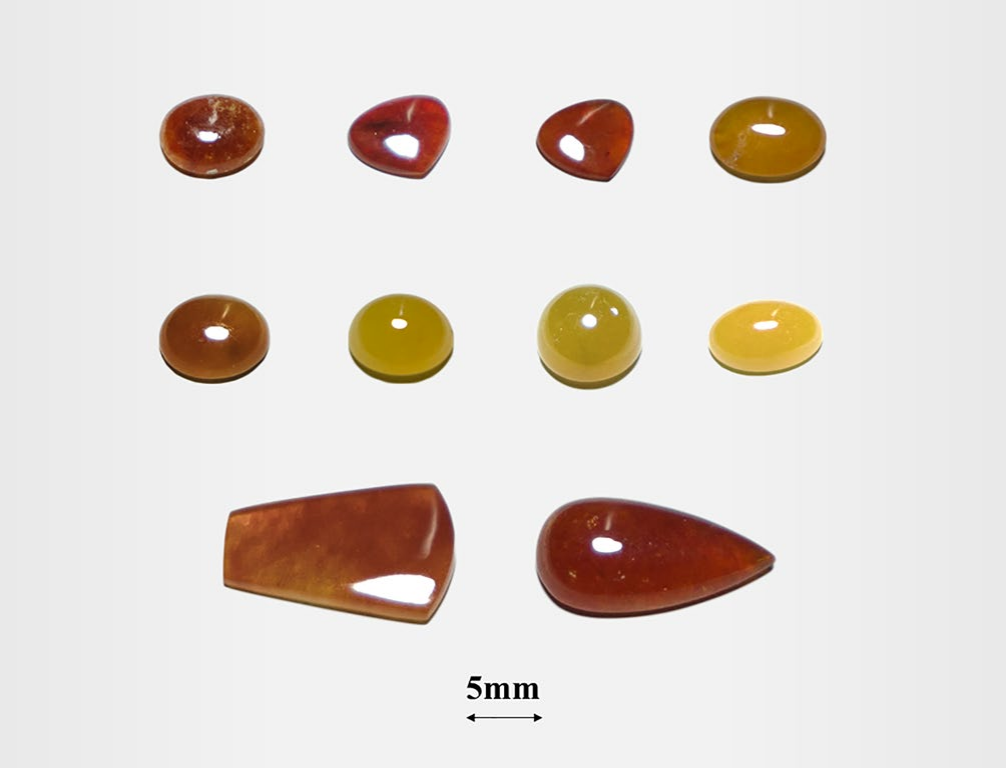
Part of the jadeite samples used in this paper.

A total of 24 Burmese jadeites are selected in this article, of which six are red, eight are orange and six are yellow. The color varies from red to yellow in steps and the color distribution is even when observed by the naked eye. The shapes are droplet, flat, and oval, with an average diameter of 7–10 mm (Fig. 1). All of the samples are gem-quality and polished, purchased from the jade market in Sihui, Guangdong Province, China.

## Result and discussion

### Influence of illuminance

The 24 pieces of jadeite samples were tested in 1100–2900 lx (interval = 200 lx) respectively (Measurement Distance: 25 cm, Exposure Time: 12,000 μs); The China national standard GB/T 26189-2010 “Lighting of Indoor Workplaces” suggests that the maintained illuminance (the lowest average illuminance on the work surface) of indoor jewelry manufacture and precious gemstone operation should be 1500 lx^32^.

**Figure 2:**
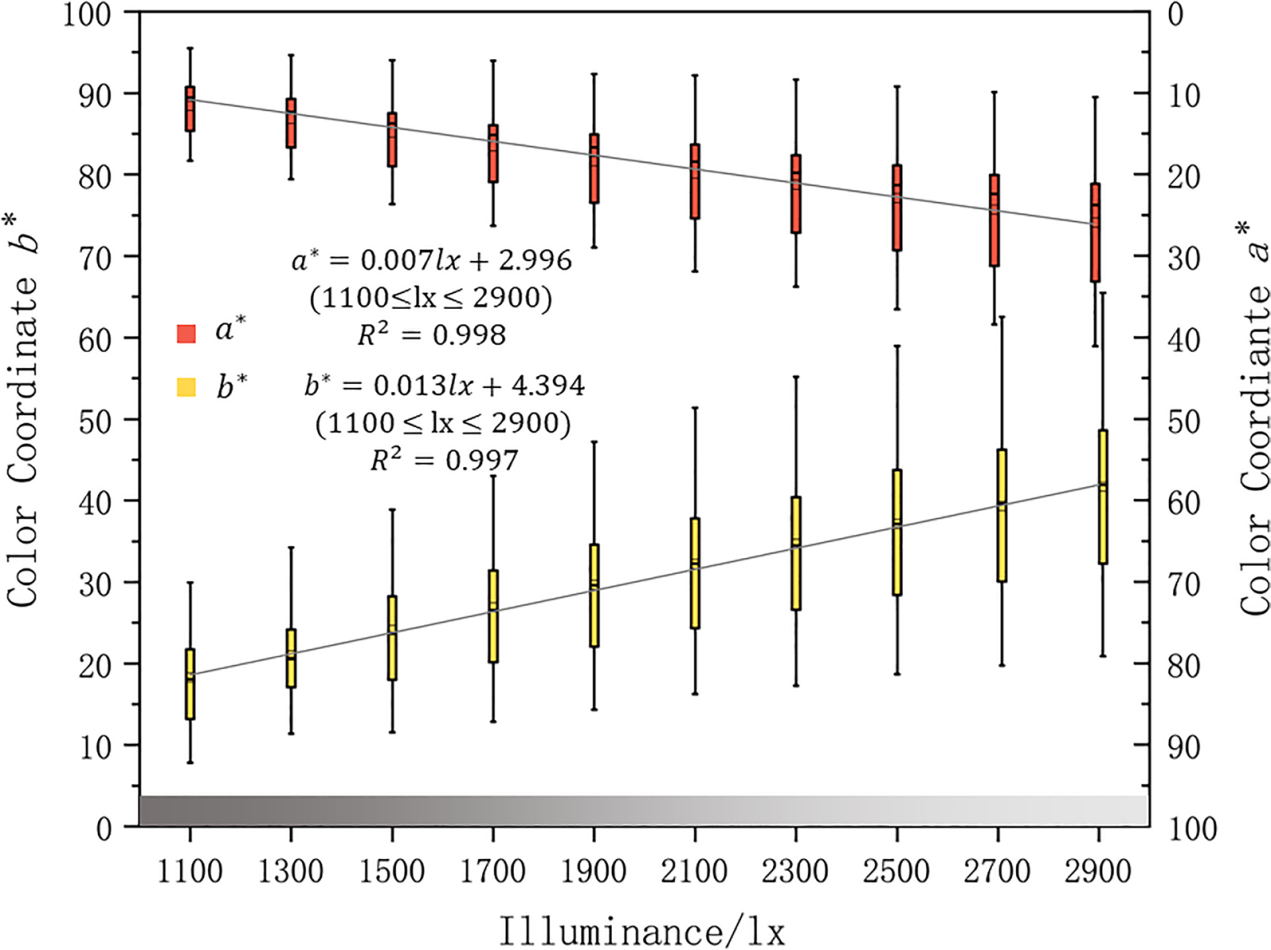
The highly positive correlation between illuminance and colorimetric coordinate ${a}^{*}$ and ${b}^{*}$.

The correlations between colorimetric coordinate ${a}^{*}{b}^{*}$ and illuminance is shown in Fig. 2. The increment of ${b}^{*}$ is more than ${a}^{*}$ with the increase of illuminance, with a fitting coefficient of 0.013 (R^2^ = 0.998) and 0.007 (R^2^ = 0.997) respectively.

**Figure 3:**
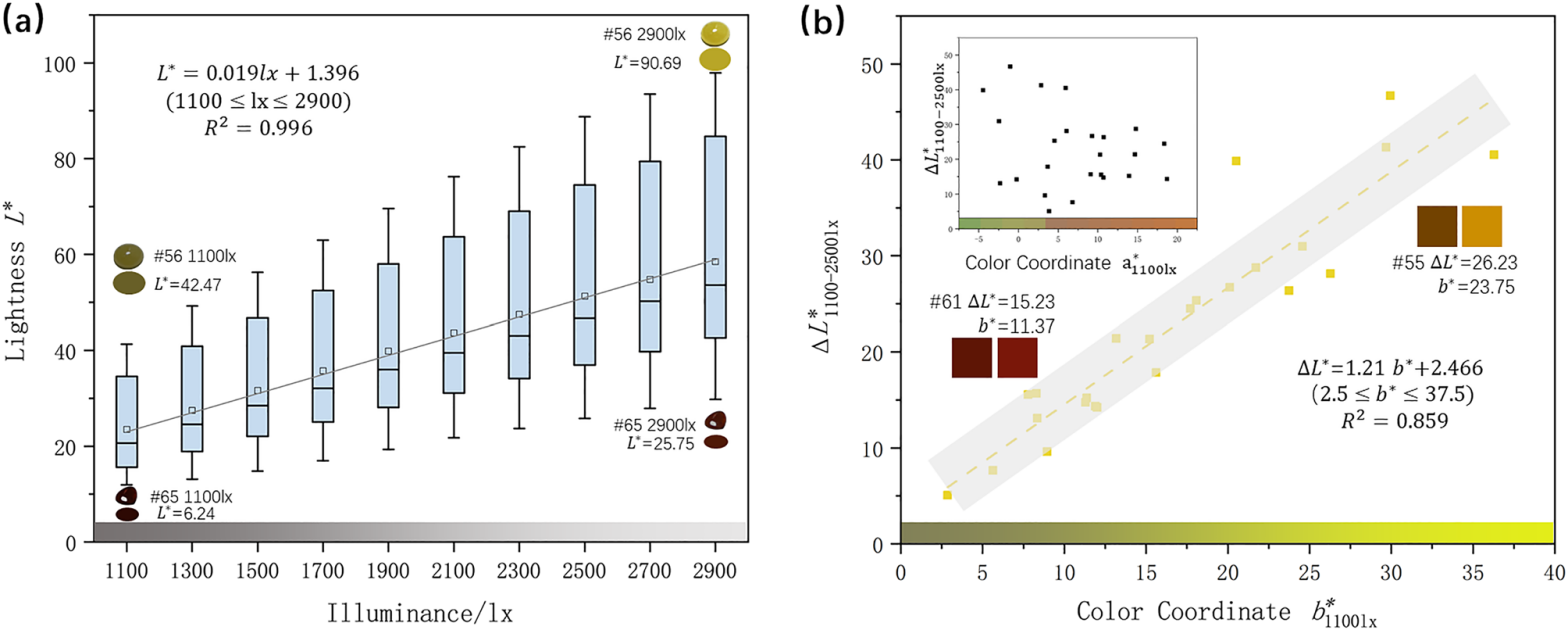
(**a**) With the rise of illuminance, the lightness ${L}^{*}$ of jadeite increases. Each box represents the distribution of the lightness of all samples at the same illuminance level. (**b**) A highly positive correlation between ${\Delta L}_{1100-2500\text{lx}}^{*}$ and color coordinate ${b}_{1100\text{lx}}^{*}$. The color boxes are the simulated color of the sample under 1100 and 2500 lx.

As shown in Fig. 3a, with the gradual increase of illuminance, the lightness ${L}^{*}$ of jadeite increases significantly and the mean lightness change from 1100 to 2900 lx is 27.81, which is the same as the observation by the naked eye. By comparing the lightness of jadeite with different hues, it is found that the lightness of yellow jadeite ($h^\circ$∈ (80,110)) has a greater change, besides, it is found that there is a high positive correlation between colorimetric coordinate ${b}_{1100 \,{\text{lx}}}^{*}$ and the change of lightness from 1100 to 2500 lx (R^2^ = 0.859) (Fig. 3b), which indicates that the lightness ${L}^{*}$ of jadeite with a higher colorimetric coordinate ${b}^{*}$ is more sensitive to the illuminance change. So those samples with a yellowish hue show a greater lightness change as the illuminance change (Fig. 3b #55 compares with #61).

**Figure 4:**
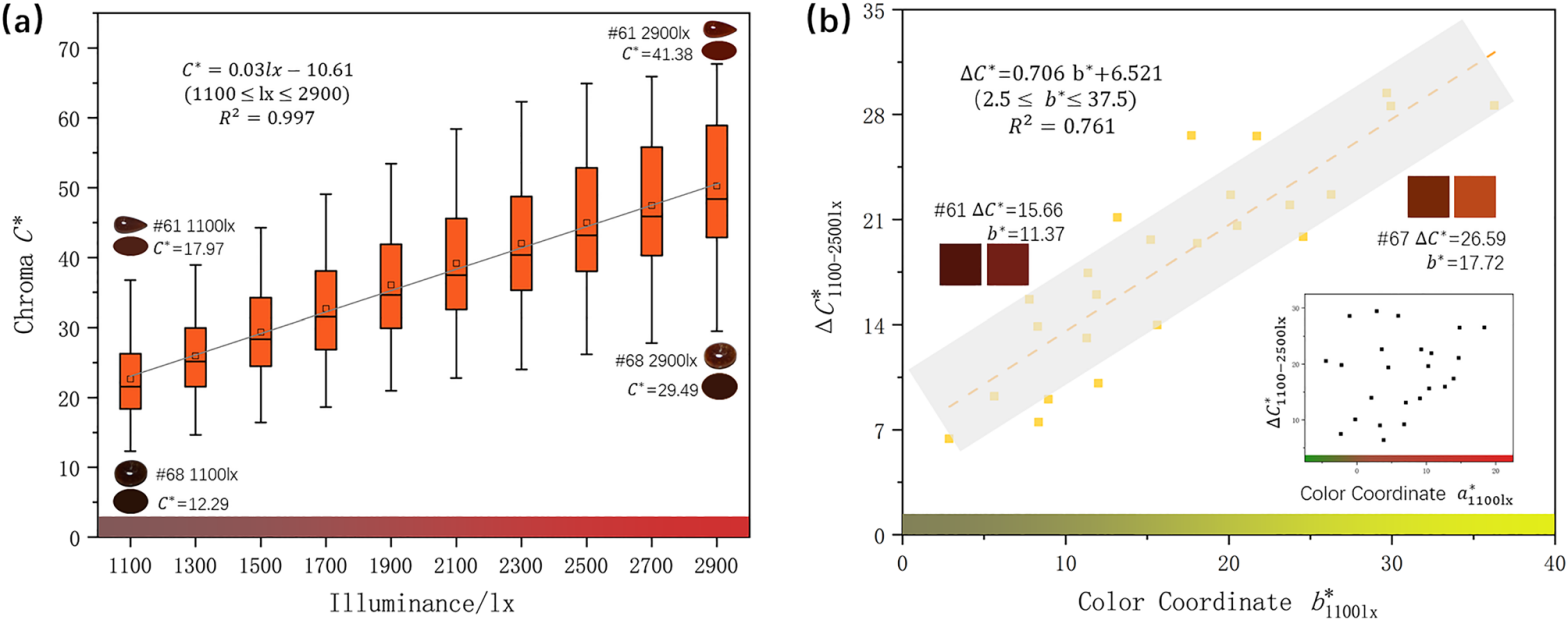
(**a**) With the rise of illuminance, the chroma of jadeite increases; (**b**) a moderate positive correlation between ${\Delta C}_{1100-2500\,{\text{lx}}}^{*}$ and color coordinate ${b}_{1100\,{\text{lx}}}^{*}$.

Figure 4a shows that the chroma ${C}^{*}$ of jadeite positively correlates to the illuminance within the range from 1100 to 2900 lx. The mean value of chroma change from 1100 to 2900 lx is 22.29 that indicating the jadeite color will be vivid as the illuminance gets higher, which is consistent with the perception that colorfulness rises as illuminance grows within the set range. As shown in Fig. 4b, a positive correlation was found between colorimetric coordinate ${b}_{1100\,{\text{lx}}}^{*}$ and ${\Delta C}_{1100-2500\,{\text{lx}}}^{*}$ (R^2^ = 0.761), combined with the result of Figs. 2 and 3, the rises in illuminance will lead to an increase in lightness ${L}^{*}$, while the colorimetric coordinate ${b}^{*}$ is positive with the change of lightness, so the chroma ${C}^{*}$ of samples with a higher ${b}^{*}$ is more sensitive with the change of illuminance (Fig. 4b #61 compares with #67).

**Figure 5:**
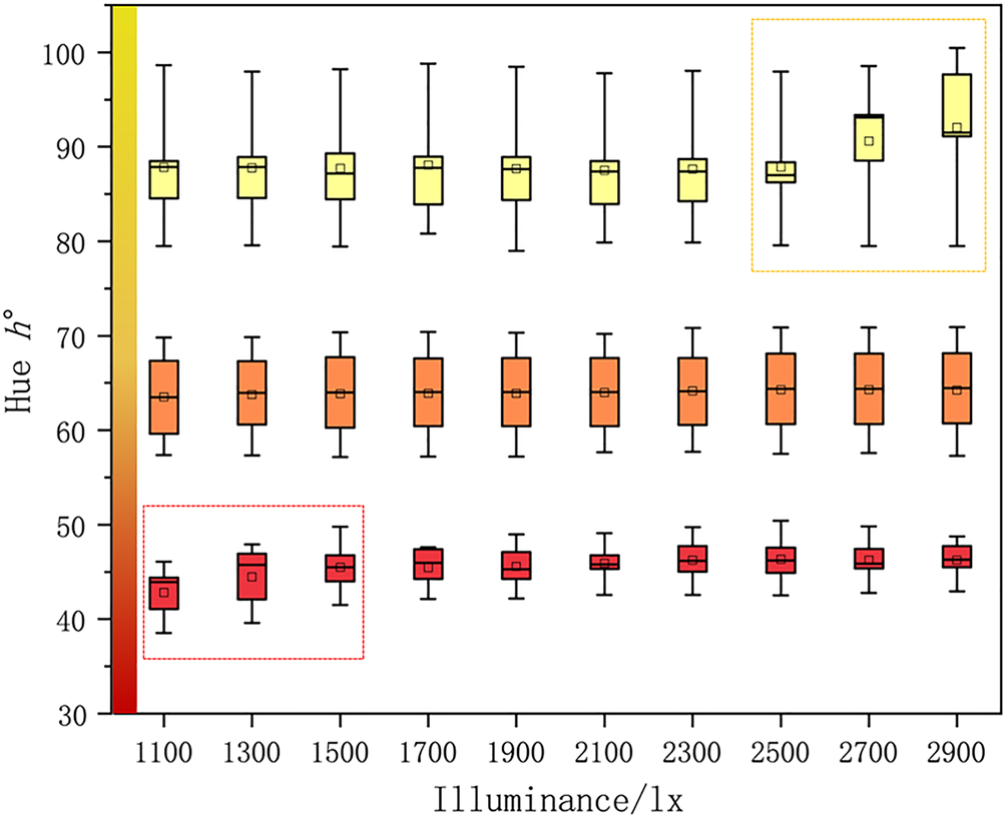
The correlation between three groups of jadeites with different hue angles h° and illuminance (group1 $h^\circ \in \left(\text{35,50}\right);$ group2 $h^\circ \in \left(\text{55,70}\right)$; group3 $h^\circ \in \left(\text{80,100}\right)$).

The mean hue h° changes (max–min) is 3.12 which indicates the color hue of jadeite slightly fluctuate as the illuminance gets higher. To better compare the hue angle variation of jadeites with different hue range, the samples were divided into 3 groups. As shown in Fig. 5, it shows that the sample whose hue angle ranges from 35° to 50° has a significant rise from 1100 to 1500 lx (red box).

The main effect of illuminance on reading the color of jadeite is to improve the lightness, chroma, and slightly hue shift, however, excessive illuminance will cause the obtained photos to be overexposed (Fig. 5 Yellow box) and read unreal data.

**Figure 6:**
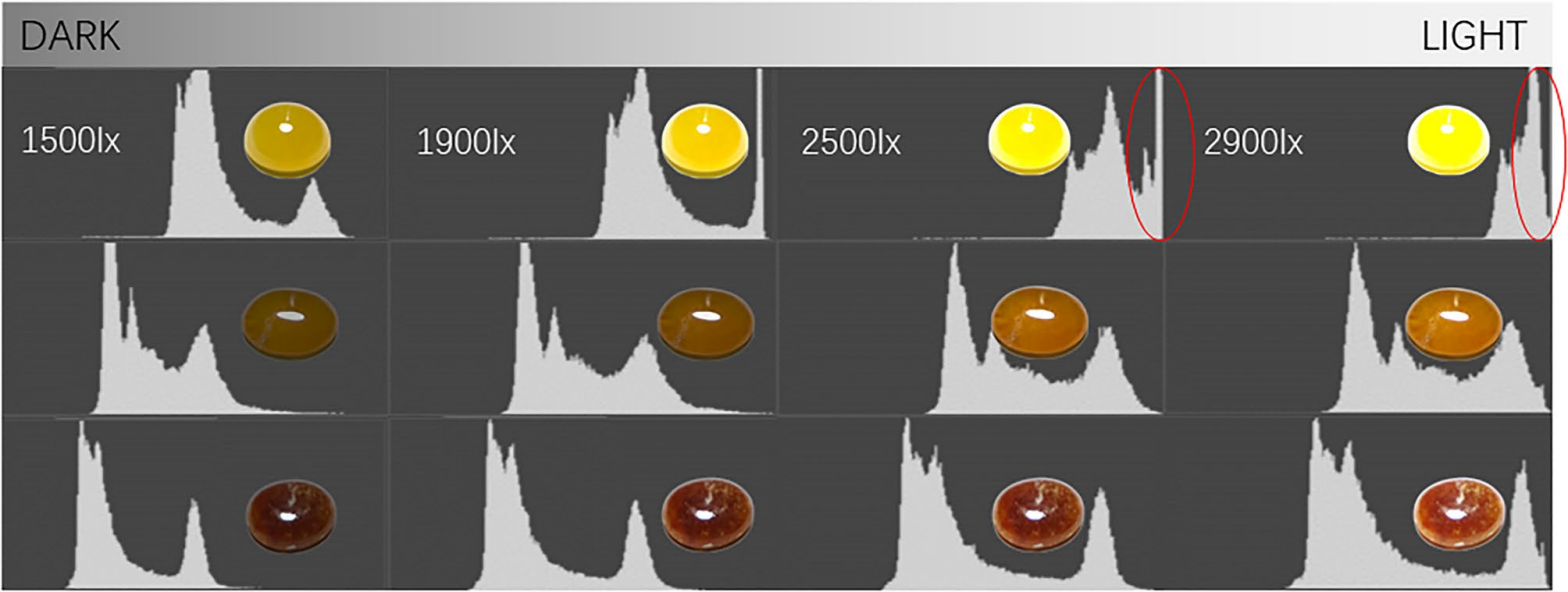
The lightness histogram of jadeite with different hue angles under different illuminance levels. All the lightness histograms were exported from PhotoShop2020.

The yellow jadeite is dominant by the colorimetric coordinate ${b}^{*}$, so its lightness and chroma show a higher sensitiveness to the change of illuminance; combined with lightness histogram, when the illuminance reaches 2500 lx and above, the lightness histogram of the yellow jadeite is occupied by the high lightness area (Fig. 6 red circle) when the histogram of red and orange jadeite shows a good uniformity, which means there is loss of color data in the yellow sample and causes the reading of unreal color. Therefore, the optimal parameters for the measurement of jadeite with different hues (red–orange–yellow) should be discussed separately. Based on the above results, the illuminance range can be further narrowed down to 1500 to 2300 lx to ensure the stability of the test.

### Influence of exposure time (E.T)

**Figure 7:**
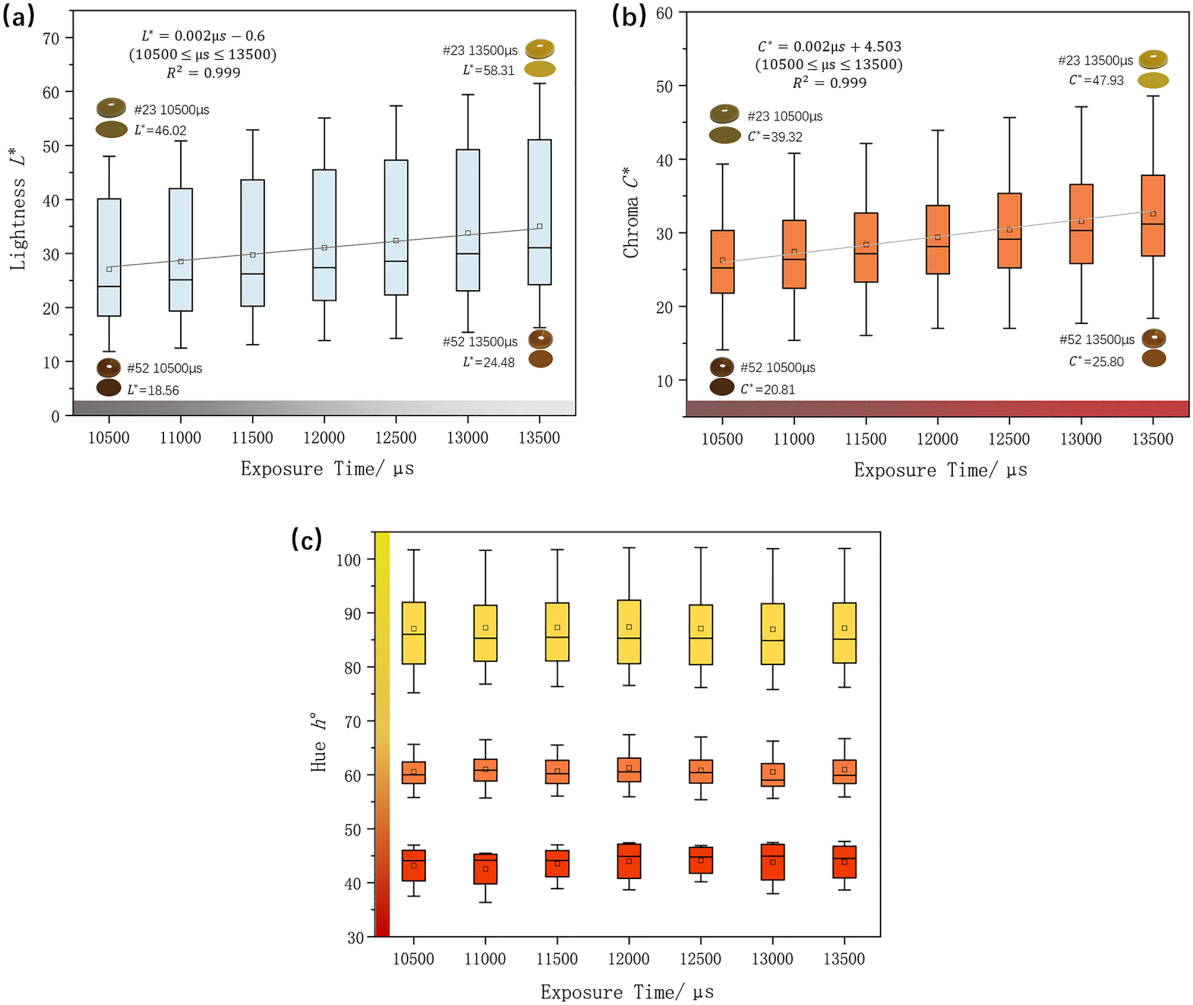
(**a**) A highly positive correlation between E.T with Lightness ${L}^{*}$ of Jadeite; (**b**) the Chroma ${C}^{*}$ is increasing when the E.T increase; (**c**) the variation of hue angle $h^\circ$ of jadeites which are divided into three groups based on hue angles.

The 14 jadeite samples were tested in 10,500–13,500 μs (interval = 500 μs; 1500 lx, 25 cm) respectively. As shown in Fig. 7a, the lightness ${L}^{*}$ of jadeite is increasing with the increase of E.T. The mean lightness change from 10,500 to 13,500 μs is 14.11, which corresponds to the objective fact that the luminous flux of the camera is increased. Figure 7b shows the Chroma ${C}^{*}$ gets higher as the E.T increases, the mean value of Chroma difference between 10,500 and 13,500 μs is 8.9. By analyzing with samples (#23 and #52), the higher ${C}_{10,500\,\upmu {\text{s}}}^{*}$ and ${L}_{10,500\,\upmu {\text{s}}}^{*}$ (the Chroma and Lightness in initial E.T value) it is, the more $\Delta {C}^{*}$ and ${\Delta L}^{*}$ will be. The E.T have little influence on the Hue $h^\circ$ of samples, with a mean change $\stackrel{-}{{\Delta h^\circ }_{max-min}}$=1.37, those jadeites with lower hue angle show a relatively greater sensitiveness than those higher (Fig. 7c). According to the similar trend of ${L}^{*} {C}^{*} h^\circ$ caused by illuminance and exposure time, the interactive influence between them is considered in the orthogonal experiment subsequently.

### Influence of measurement distance

Under the conditions of 1500 lx and 12,000 μs, the 14 samples were tested in 20–35 cm (interval = 5 cm) respectively. The mean change $\overline{{\Delta L }^{*}}=$ 0.273, $\overline{{\Delta C }^{*}}=$ 0.326, $\stackrel{-}{\Delta h^\circ }=$ 0.383, which illustrates the influence of M.D is smaller than the illuminance and exposure time. Since the size and shape of jadeite in the market are very variable, the stability of the color measurement test can be simulated by changing the measurement distance. According to the results, it can be found that the digital image system can provide good stability under the set lighting and background conditions.

### Optimal parameter for jadeite-red by DIS

**Figure 8:**
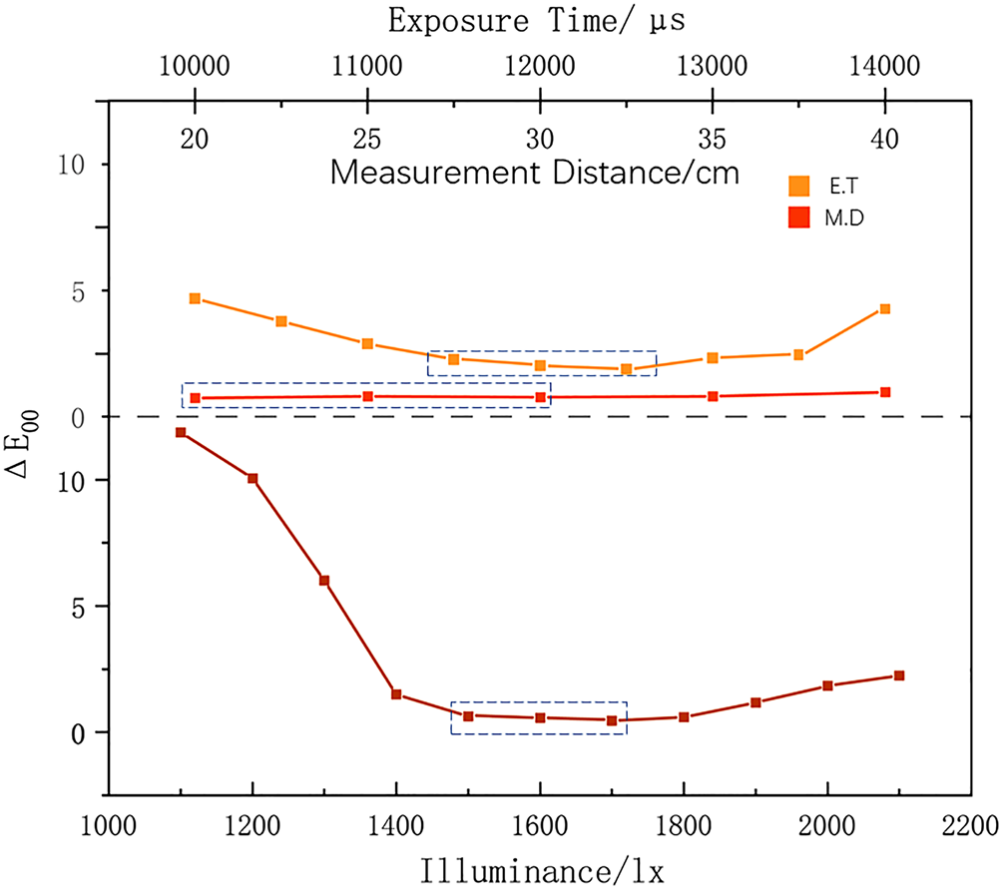
The pre-test result for the orthogonal design, which shows the selected 3 levels for each factor with a black box. ${\Delta E}_{00}$ is the color difference between data from DIS and date from photo calibration calculated by Eq. (9). The box is the range for the subsequent Orthogonal Design, the reason for the selected levels is based on the lowest ${\Delta E}_{00}$ and China national standard GB/T 26189-2010 “Lighting of Indoor Workplaces” and GB/T 23885-2009 “Jadeite grading”. From top to bottom: Exposure Time, Measurement Distance, Illuminance.

Six red jadeites from the tested samples were chosen for this chapter. According to the pre-test shown in Fig. 8, three levels for each factor were selected for the subsequent orthogonal experiment. The color difference was used here to measure the degree of applicability of the combination of test parameters, the smaller the $\overline{{\Sigma \Delta E }_{00}}$, the better the combination.

**Figure 9:**
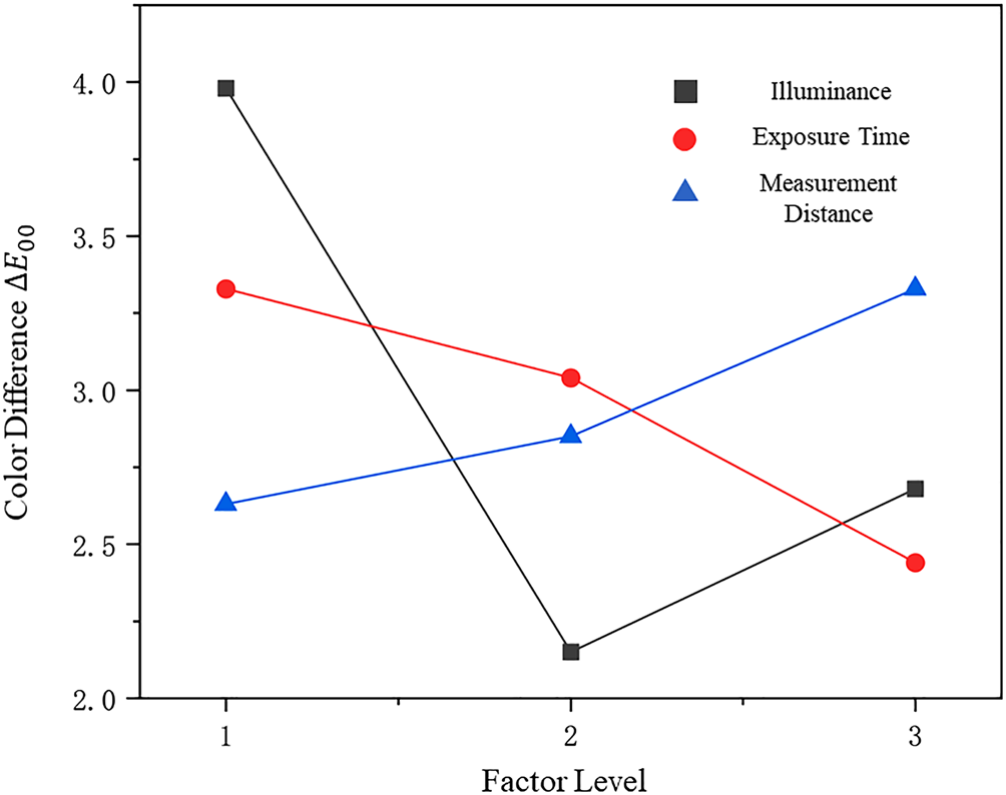
The change trend chart of factor and level.

As is shown in Table 1, L18 (36) is selected for the 3-level 3-factor experiment with 2 interactions (A × B). According to the result of range analysis, R1 > R2 > R5 indicates the order of degree of influence of the experimental factors on the result: Illuminance > Exposure Time > Measurement Distance. For the result of illuminance, because it is based on the average color difference ΔE00—the smaller it is, the better the result is—k2 < k3 < k1 represents the most suitable level is 1600 lx. And so on the best measurement distance is 25 cm, and exposure time is 1250,0 μs (Fig. 9).

**Table 1:** Orthogonal design.

Num	Illuminance (A)	E.T (B)	A × B	A × B	M.D	Error	$\overline{{\Sigma \Delta E }_{00}}$
1	1500	11,500	1	1	20	1	2.46
2	1500	11,500	2	2	30	3	2.79
3	1500	12,000	1	3	30	2	2.03
4	1500	12,000	3	1	25	3	2.06
5	1500	12,500	2	3	25	1	1.31
6	1500	12,500	3	2	20	2	1.28
7	1600	11,500	1	3	25	3	1.57
8	1600	11,500	3	1	30	2	1.33
9	1600	12,000	2	2	25	2	1.03
10	1600	12,000	3	3	20	1	1.06
11	1600	12,500	1	2	30	1	0.73
12	1600	12,500	2	1	20	3	0.73
13	1700	11,500	2	3	20	2	0.91
14	1700	11,500	3	2	25	1	0.94
15	1700	12,000	1	2	20	3	1.45
16	1700	12,000	2	1	30	1	1.48
17	1700	12,500	1	1	25	2	1.63
18	1700	12,500	3	3	30	3	1.64
K_1_	11.93	10	9.87	9.69	7.89	7.98	T = 26.43
K_2_	6.45	9.11	8.25	8.22	8.54	8.21	
K_3_	8.05	7.32	8.31	8.52	10	10.24	
k_1_	3.98	3.33	3.29	3.23	2.63	2.66	
k_2_	2.15	3.04	2.75	2.74	2.85	2.74	
k_3_	2.68	2.44	2.77	2.84	3.33	3.41	
R_j_	1.83	0.89	0.54	0.49	0.70	0.75	

K_i_ is the sum of the color difference ΔE of the same level of the column; k_i_ is the mean of K_i_; Rj is the Range.

ANOVA can help distinguish between data fluctuations caused by changes in experimental conditions and data fluctuations caused by experimental errors. Again, the results of the ANOVA can further verify the conclusions of the above range analysis. The ${SS}_{T}$ indicates the sum of squared deviations where $n$ represents the total number of experiments, ${y}_{i}$ represents the result of each experiment, and SS_T_ is calculated by:

1 $$S{S}_{T}={{\sum }_{i=1}^{n}\left({y}_{i}-\overline{y}\right)}^{2}={\sum }_{i=1}^{n}{y}_{i}^{2}-\frac{1}{n}{\left({\sum }_{i=1}^{n}{y}_{i}\right)}^{2}.$$

The sum of squared deviations of each column SS_j_ as:

2 $$S{S}_{j}=\frac{r}{n}\left({\sum }_{i=1}^{r}{K}_{i}^{2}\right)-\frac{{T}^{2}}{n},$$

where T is the sum of $\stackrel{-}{{\Sigma {\Delta}E}_{00}}$.

The degrees of freedom of each factor ${f}_{A}$ and the total degrees of freedom ${f}_{\text{total}}$ are calculated as follows:

3 $${f}_{A}=m-1,$$

4 $${f}_{total}=n-1,$$

where m represents the number of levels.

According to the sum of square deviations of each factor ${V}_{A}$, the statistic $F$ value can be calculated as:

5 $${V}_{A}=\frac{{S}_{A}}{{f}_{A}},$$

6 $${F}_{A}=\frac{{V}_{A}}{{V}_{MS}}=\frac{\frac{{S}_{A}}{{f}_{A}}}{\frac{{s}_{MS}}{{f}_{MS}}}.$$

According to the results of the ANOVA (Table 2), the conclusions of the range analysis can be verified. The order of the degree of factors that influence the DIS is Illuminance (A) > Exposure Time (B) > A × B > Measurement Distance. Illumination has a very significant influence on the DIS, and the k_2_ level is the best; the effect of exposure time have some influence, and the result shows that the k_3_ level is the best; the effect of measurement distance is not obvious, and the level with the lowest color difference k1 can still be selected for the result; the interaction between illuminance and exposure time is not obvious, according to the analysis of the interactive influence, it is found that the A2B3 combination is the best level (Table 3), which corresponds to 1600 lx, 12500 μs; finally, the optimal parameter level combination for the red jadeite color reading by the DIS is 1600 lx, 12,500 μs, and 25 cm. In the future, more test samples can be added to modify the optimized measurement conditions, and based on this basis, measurement parameters can be obtained for other colors of jadeite.

**Table 2:** Analysis of variance.

Factors	S_A_	f_A_	V_A_	F	F distribution	Sig
Illuminance (A)	2.64	2	1.32	18.0	F_0.10_(2,6) = 3.11	***
E.T (B)	0.62	2	0.31	4.2	F_0.05_(2,6) = 4.46	*
A × B	0.48	4	0.12	1.6	F_0.01_(2,6) = 8.65	–
M.D	0.2	2	0.1	1.4		–
Error	0.516	7	0.0737			
Total	4.467	17				

Compare the F value with the F distribution. If F is greater than F_0.01_(2,6), the result is very significant (***), greater than F_0.05_(2,6) is significant, greater than F_0.10_(2,6) represents to have some influence (*), otherwise (-) deemed to have no significant impact.

**Table 3:** Interactive influence between illuminance and E.T.

	A1	A2	A3
B1	2.63	1.45	0.93
B2	2.05	1.05	1.47
B3	1.3	0.73	1.64

### Verification experiments

10 members observed all the simulated color blocks then say “yes” to the test result, which means every scheme can represent the average color of jadeite without comparison by different scheme. As shown in the Table 4, the scheme A was graded as 2 (low) with frequency 13.89% while the scheme C with 1.39% and B with 0.00%. Although the frequency of all schemes with a score greater than 3 was higher than 50%, the scheme B got 4 and 5 points more frequently than the others, which indicates that the parameters for DIS can better restore the color of jadeite red.

**Table 4:** Result of validation group.

Scheme	Num of test	Frequency					Total (%)
		5 (%)	4 (%)	3 (%)	2 (%)	1 (%)	
A	72	0.00	13.89	72.22	13.89	0.00	100
B	72	73.61	16.67	9.72	0.00	0.00	100
C	72	23.61	54.17	20.83	1.39	0.00	100

A represent 1700 lx, 11,500 μs, 25 cm; B represents 1600 lx, 12,500 μs, 25 cm; C represents 1600 lx, 12,000 μs, 25 cm. Total equals 100% indicates that all members consider the simulated color to be representative of the average sample color (in the absence of scheme comparison).

## Conclusion

DIS is a non-contact color measurement system that could help researchers better study unevenly distributed colored gems. The system includes circumstance factors such as light source intensity (illuminance), exposure time, measurement distance, etc., which will affect the color reading of jadeite. Under the single factor experiment method: 1. The influence of illuminance is mainly to improve the test results of lightness and chroma. The hue angle will fluctuate as the illuminance rise. 2. The effect of exposure time is mainly to increase the luminous flux, thereby improving the lightness and chroma of jadeite, and the overall change trend is consistent with the change in illuminance; 3. The DIS shows good stability on the change of measurement distance, which is used to simulate the measurement on jadeites with different sizes and shapes.

Based on the results of single-factor experiments, a three factors three levels orthogonal experiment with two interactive effects was designed. The order of the degree of influence obtained by range analysis and ANOVA is illuminance, exposure time, measurement distance (from high to low). Combined with the result, the optimal color measurement parameters of jadeite-red for DIS are 1600 lx, 12,500 μs, 25 cm; this provides a new case and theoretical support for the future application of DIS on reading the color of gemstones and the establishment of a gem-color database.

## Methods

### Color space

The CIE (International Commission de l’Éclairage) 1976 ${L}^{*}{a}^{*}{b}^{*}$ uniform color space has been widely used in the color evaluation of various colored gemstones; compared to the CIE1931 color space, the geometric distance between two given colors is inconsistent with human visual perception^33^. CIE1976 ${L}^{*}{a}^{*}{b}^{*}$ uniform color space has two advantages: (1) good color uniformity and (2) conform to the subjective law that the visual color difference in the yellow-blue direction is bigger than that in the red-green direction. This color system comprises color coordinates ${a}^{*}$, ${b}^{*}$ and Lightness ${L}^{*}$. Chroma ${C}^{*}$ and Hue angle $h^\circ$ can be calculated based on ${a}^{*}$ and ${b}^{*}$:

7 $${C}^{*}=\sqrt{{a}^{*2}+{b}^{*2},}$$

8 $$h^\circ =\mathit{arctan}\left(\frac{{b}^{*}}{{a}^{*}}\right).$$

### Color difference formula

To explore the optimal parameter combination of the Digital Image System (DIS) for measuring jadeite, this paper chooses the CIE DE2000 formula to analyze the results of the measured samples. The DE2000 formula is experimentally obtained based on the color difference formulas such as CIE LAB and CIE 94, which is more accurate and more computational:

9 $$\Delta {E}_{00}={\left[{\left(\frac{\Delta L{^{\prime}}}{{K}_{L}{S}_{L}}\right)}^{2}+{\left(\frac{\Delta C{^{\prime}}}{{K}_{C}{S}_{C}}\right)}^{2}+{\left(\frac{\Delta H{^{\prime}}}{{K}_{H}{S}_{H}}\right)}^{2}+{R}_{T}\left(\frac{\Delta C{^{\prime}}}{{K}_{C}{S}_{C}}\right)\left(\frac{\Delta H{^{\prime}}}{{K}_{H}{S}_{H}}\right)\right]}^\frac{1}{2},$$

where $\Delta {L}^{{{\prime}}}$, $\Delta {C}^{{{\prime}}}$, $\Delta {H}^{{{\prime}}}$ represent the difference in lightness, chroma, and hue angle respectively (between the DIS data and photo calibrated data). The weight functions S_L_, S_C_, and S_H_ are used to calibrate the uniformity of the color space, and the function ${R}_{T}$ is used to correct the deflection of the color tolerance ellipse in the blue area; the Parameters ${K}_{L},{K}_{C},{K}_{H}$ are to correct the experimental conditions, in gemology, where the parameter combination is usually set as (1:1:1) to provide better perceptibility.

The color difference is calculated at the “colortell.com” (developed by ColorTell Tech CO., LTD, Beijing, China) color management website by importing digital imaging system data (samples) and calibrated photo data (standards), after which the color data is calculated by the Eq. (9).

### Digital imaging system (DIS)

**Figure 10:**
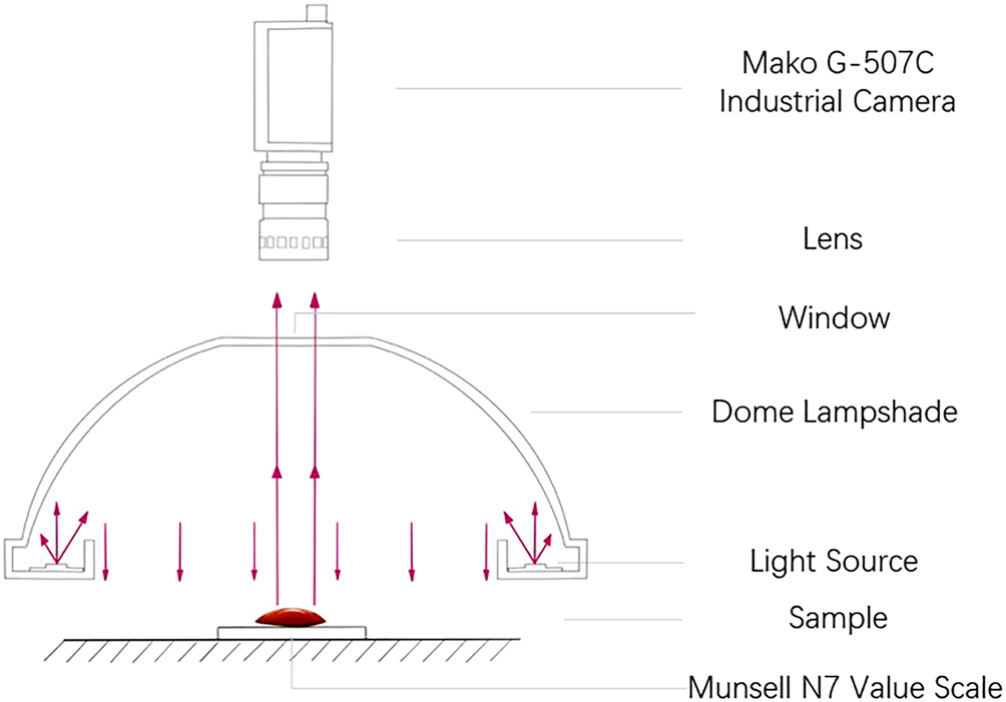
The DIS experimental device.

**Figure 11:**
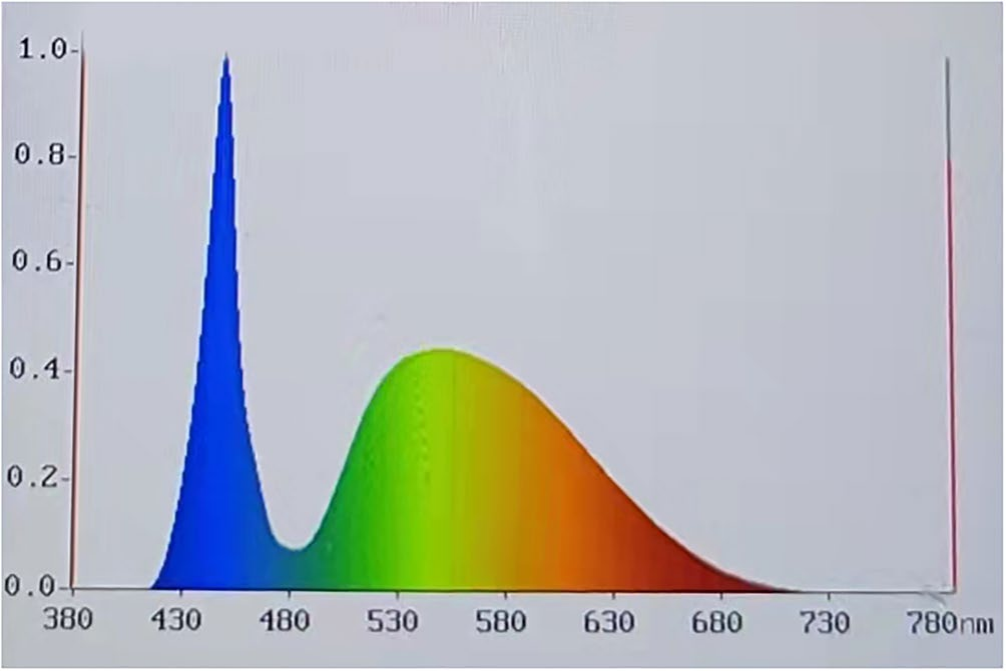
Actual measured spectral power distribution.

The color of jadeite was measured by the Digital imaging system under the same Munsell N7 background (Fig. 10). The images were captured by using a Mako G-507C industrial camera (Allied Vision Technologies, Stadtroda, Germany) equipped with a CMOS-type Sony IMX264 sensor with a size of type 2/3. The resolution of the camera is 2464 (horizontal) × 2056 (vertical), it has a global shutter, a 50 mm focal length, and an aperture factor of 1.4. The dome lampshade is a type of shadowless dome light source with a color temperature of 6326 K (Color Rendering index is 90, the power distribution is shown in Fig. 11), which is positioned 10 cm above the sample. To better read the color of the sample, a special diffuse reflection material is used inside the lampshade. So that the light can be evenly irradiated on the surface of the sample. A Konica Minolta CL-200 Color Illuminometer (Tokyo, Japan) served to measure the environmental illuminance of the measuring position.

The color reading was processed by the CKVisionBuilder V3.0 software (developed by Shenzhen CKVision Machine Vision Technology Co., Ltd., Shenzhen, China). The color conversion algorithm is encapsulated in the supporting CKVisionSDK 3.0. The user can select different types of tools to read the color of the specified area in the picture, and the software can convert the sRGB value of the selected area to the ${L}^{*}{a}^{*}{b}^{*}$ value. Three areas were chosen to calculate the average color parameters for each sample.

**Figure 12:**
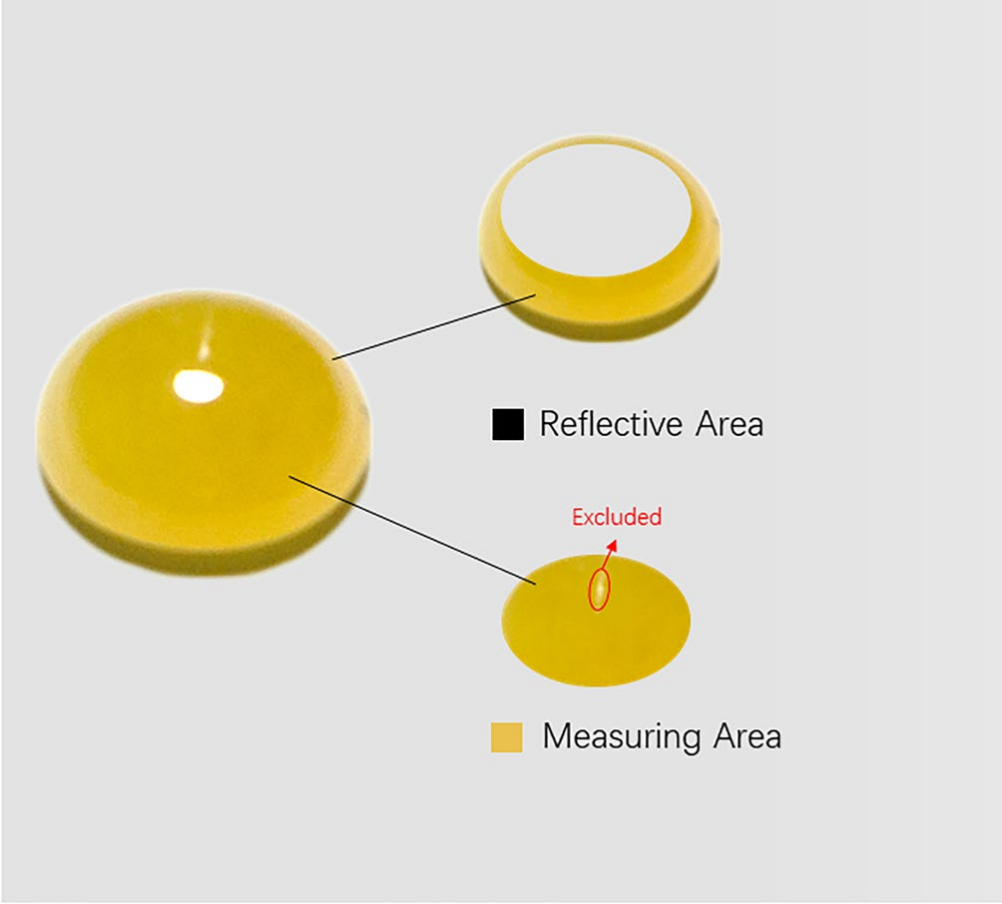
The reflective area with strong reflection and inclusion in the measuring area should be avoided during the color reading process.

All the jadeite samples were well polished and showed a high quality “glassy luster” because of their high refractive index (1.66). To prevent the influence of surface reflection on color measurement, we excluded the reflective areas from the color reading, selected three uniformly colored locations in the measuring area, measured the color, and took the average value as the test result (Fig. 12). Some samples containing black or white mineral inclusions were excluded from the measuring area.

### Photograph calibration

**Figure 13:**
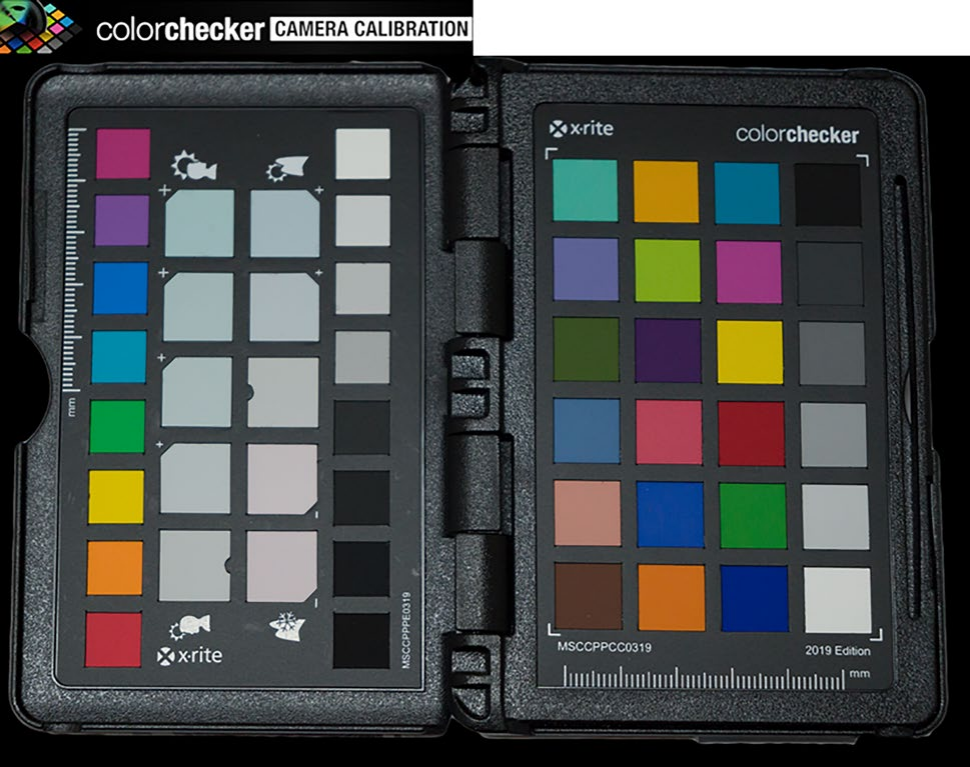
X-rite Colorchecker passport.

The Nikon Z5 (Nikon, Tokyo, Japan) equipped with a CMOS-type sensor is used to take photos of X-rite Color Checker Passport (X-rite, Shanghai, China) (Fig. 13) and jadeite samples under the standard D65 light source, and all the samples are placed at the Munsell N7 neutral grey background.

**Figure 14:**
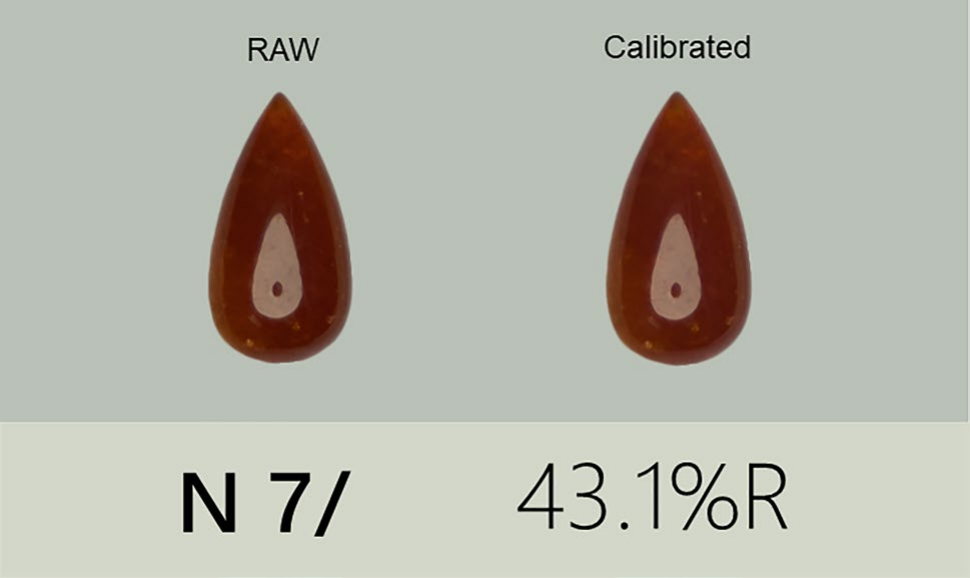
The pictures of the sample (RAW and Calibrated) were placed on the Munsell N7 value scale.

X-rite Color Checker Passport contains a set of three-Color Checker photography targets^34^, a desktop camera calibration application, and an Adobe Photoshop Lightroom camera calibration plug-in to create a DNG profile that reflects specific camera, lens, and lighting conditions to process the calibration of the RAW photo and restore the true color of the jadeite. Each photo was processed in Photoshop 2020, using the established environment DNG feature file for correction (Fig. 14), operation steps as follow:

1. Take the picture of ColorChecker and samples under the set circumstance, the output format is RAW.
2. Import the picture including ColorChecker into “ColorChecker Camera Calibration” (developed by X-rite, Shanghai, China) to create a DNG profile.
3. Open picture of samples through Photoshop2020, then set the DNG profile as the configuration file.

### Single factor experiment

This paper applied the single factor experiment method to test the three main influencing factors of the DIS (Illuminance, Exposure Time (E.T), Measurement Distance (M.D)). While one of the factors is regarded as a variable, the other two factors are regarded as constants, for example, when testing the influence of Illuminance, the experimental conditions were set as 1. Illuminance: 1100 lx, 1300 lx, 1500 lx, 1700 lx, 1900 lx, 2100 lx, 2300 lx, 2500 lx, 2700 lx, 2900 lx; 2. Exposure Time: 12,000 μs; 3. Measurement Distance:25 cm. To explore the influence of variables on the experimental results.

### Orthogonal experiment design

An L_18_ (3^6^) Orthogonal table was selected for this paper, with 3 factors 3 levels, and 2 interactives. A blank column was set to consider the error. And the average color difference of each combination of experimental conditions is assigned to determine the feasibility of the experimental result. The ANOVA was set to investigate the degree of influence of experimental conditions on experimental results.

### Validation group

**Figure 15:**
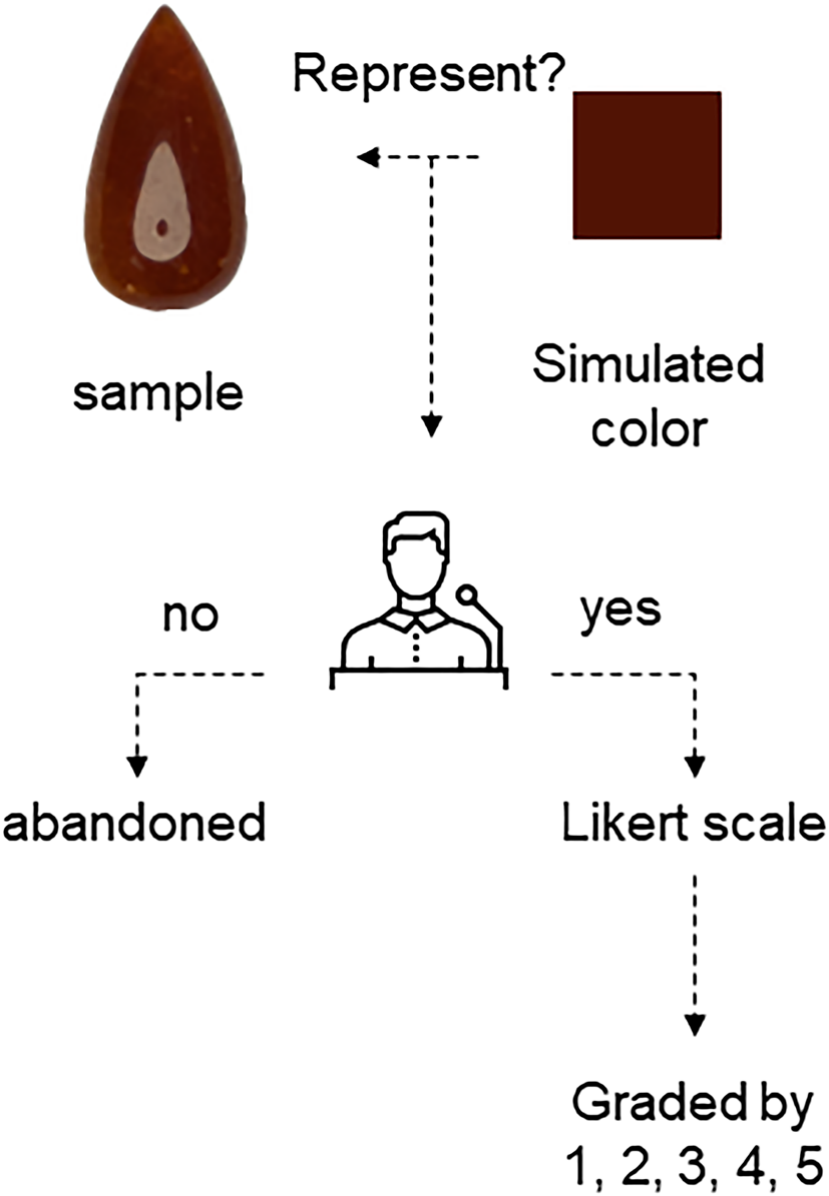
The process of validation.

10 people were chosen to participate in this experiment, two of whom own certificate of The Gemmological Association of Great Britain. They ranged in age from 20 to 60 years and were in good health. The members observe the simulated color boxs exported by different parameters (shown on the 15.6 inchs screem LGD05C0 which is produced by LG Display, Korea) and physical samples (In the northern hemisphere at noon daylight), they have 10 s to decide whether the simulated color can represent the sample by “yes” or “no”. If the answer is “yes”, then they will give a grading point from 1 to 5 on the five-point Likert scale: 1 is “very low”, 2 is “low”, 3 is “moderate”, 4 is “high”, 5 is “very high” (Fig. 15).

## Data Availability

Data underlying the results presented in this paper are not publicly available at this time but may be obtained from the authors upon reasonable request.
